# Utilization of 3-Month Yoga Program for Adults at High Risk for Type 2 Diabetes: A Pilot Study

**DOI:** 10.1093/ecam/nep117

**Published:** 2011-01-09

**Authors:** Kyeongra Yang, Lisa M. Bernardo, Susan M. Sereika, Molly B. Conroy, Judy Balk, Lora E. Burke

**Affiliations:** ^1^School of Nursing, University of Pittsburgh, Pittsburgh, PA 15261, USA; ^2^School of Medicine, University of Pittsburgh, Pittsburgh, PA 15261, USA

## Abstract

Various modes of physical activity, combined with dieting, have been widely recommended to prevent or delay type 2 diabetes. Among these, yoga holds promise for reducing risk factors for type 2 diabetes by promoting weight loss, improving glucose levels and reducing blood pressure and lipid levels. This pilot study aimed to assess the feasibility of implementing a 12-week yoga program among adults at high risk for type 2 diabetes. Twenty-three adults (19 Whites and 4 non-Whites) were randomly assigned to the yoga intervention group or the educational group. The yoga group participated in a 3-month yoga intervention with sessions twice per week and the educational group received general health educational materials every 2 weeks. All participants completed questionnaires and had blood tests at baseline and at the end of 3 months. Effect sizes were reported to summarize the efficacy of the intervention. All participants assigned to the yoga intervention completed the yoga program without complication and expressed high satisfaction with the program (99.2%). Their yoga session attendance ranged from 58.3 to 100%. Compared with the education group, the yoga group experienced improvements in weight, blood pressure, insulin, triglycerides and exercise self-efficacy indicated by small to large effect sizes. This preliminary study indicates that a yoga program would be a possible risk reduction option for adults at high risk for type 2 diabetes. In addition, yoga holds promise as an approach to reducing cardiometabolic risk factors and increasing exercise self-efficacy for this group.

## 1. Introduction

Type 2 diabetes affects more than 21 million Americans [1], and the death rate from diabetes has increased 45% since 1987 [2]. One important regimen for people with diabetes and for those at risk for developing diabetes is engagement in appropriate physical activity. The beneficial effects of physical activity typically include reductions in glucose level, body weight, blood pressure (BP) and cholesterol [3–6]. As a particular form of physical activity, yoga programs using various physical postures have been shown to benefit individuals with a wide range of health conditions including hypertension [7] and diabetes [8]. Yoga appears to be especially effective in reducing BP, glucose and cholesterol [7–11]. For example, yoga practice was effective in lowering BP in healthy people [9], people with hypertension [7, 8] and people with metabolic syndrome [11]. Adults with chronic diseases including hypertension, coronary artery disease and diabetes had significantly lower glucose and cholesterol levels after short-term, intensive yoga practice (3-4 h per day for 8 days) [8]. Yoga also improved exercise tolerance by significantly reducing exercise-induced cardiovascular changes (e.g., heart rate and BP) [12]. Deep relaxation, a unique part of a yoga program, relaxes the sympathetic nervous system [13] and helps with physiological stress reduction [14, 15]. Physiological stress itself is related to metabolic disease, including diabetes.

The practice of yoga generally includes meditation, relaxation, breathing exercises and various physical postures [16]. Between 1997 and 2007, the number of yoga practitioners significantly increased in the USA [17]. The 2007 National Health Interview Survey [17] showed that 6.1% of US adults practiced yoga in the months immediately prior to the survey in 2007, compared with 3.7% in 1997 and 5% in 2002 [18]. In addition, adults participating in a yoga intervention found that yoga was easily learned and performed [19]. Once learned, yoga can be practiced at any time on an individual basis, thus reducing common barriers to physical activity such as time conflicts and poor weather.

Self-efficacy, the degree of confidence one has in the ability to perform a behavior, is regarded as one of the primary factors that is influential in achieving behavior change [20, 21]. Research suggests that yoga participation and mastery could improve the degree of exercise self-efficacy and perhaps lead to long-term adherence to physical activity and its attendant benefits [22]. In Oleshansky's 2004 study of American adults aged 29–40 years [22], hatha yoga practitioners showed higher levels of self-efficacy for physical activity, including yoga, than non-practitioners.In spite of its growing acceptability and known positive effects on physiological variables, yoga has not been widely adopted as part of a regimen to prevent type 2 diabetes. It is not clear, therefore, whether individuals at risk for type 2 diabetes might benefit from the regular practice of yoga. The purposes of this study were to assess the feasibility of implementing a 12-week yoga program among adults at high risk for type 2 diabetes, to begin examining whether yoga improves cardiometabolic risk factors (i.e., BP, lipid and blood glucose levels, and body weight), and to examine the effect of yoga on exercise self-efficacy.

## 2. Methods

### 2.1. Design and Setting

**Figure 1:**
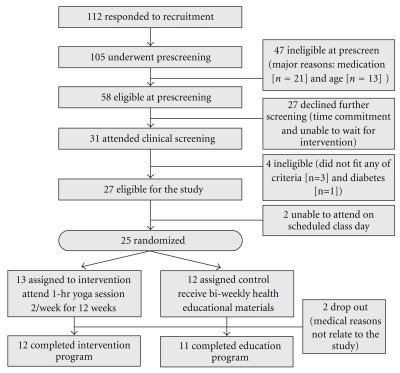
Screening and enrollment flow chart.

This study used a two-group, randomized and controlled trial design. After Human Subjects Committee's approval was obtained, participants were recruited via automated voice mail messages that were delivered to university employees, flyers posted in hospital outpatient waiting rooms and advertisements in local newspapers. To be eligible, participants had to be between 45 and 65 years of age, non-exercisers (no more than 30 min twice per week) for the previous 12 months, had a family history of type 2 diabetes (first- or second-degree relative), and at least one of the following cardiometabolic risk factors: impaired fasting glucose (FPG > 110 but <125 mg/dl); prehypertension (systolic BP/diastolic BP: 120–139/80–89 mmHg); overweight/obese [body mass index (BMI) >25 but <45 kg/m^2^]; or abnormal level of cholesterol (total cholesterol >200 mg/dl). Persons who were pregnant, those who had used any drug to reduce their blood cholesterol level, BP or glucose, or those who had a physical disability that would limit their ability to practice yoga were excluded from the study. After respondents to recruitment announcements were screened by telephone for eligibility (age, exercise status, family history of diabetes, willingness to be randomized, pregnancy status, medication history and physical limitations), we confirmed their eligibility with additional screening tests for glucose, BP, cholesterol level and BMI at the Montefiore University Hospital Clinical and Translational Research Center (MUH-CTRC). To avoid any disappointment with group assignment, we fully informed individuals of the randomization process when they first contacted the Project Office for the initial telephone screening. A copy of the consent form was sent for their review prior to their screening visit to the MUH-CTRC. We reviewed the study details described in the consent form with them in person and responded to their questions before they signed the consent form. Twenty-three participants (19 Whites and 4 non-Whites) were randomly assigned to a yoga intervention group or a control group (Figure 1).

### 2.2. Intervention (or Experimental Procedure)

Participants in both the intervention group and control group were asked to maintain their current activity levels during the intervention. The intervention group participated in a 3-month Vinyasa style yoga intervention program developed and led by a certified yoga instructor who is a PhD certified nurse. Vinyasa yoga is an evolving form of hatha yoga, so both yoga styles share the same basic elements, but Vinyasa is more fitness-based than traditional hatha yoga. This Vinyasa style yoga program included various physical postures (*Asanas*) such as sun-salutations, standing poses, seated/kneeling poses and counterposes. Each movement was combined with various breathing patterns of inhalation and exhalation (*Pranayamas*). All postures were modified to meet the participants' needs and to assure safety. For example, tree pose, a one-leg balance pose, was performed using a chair or wall for support when a participant had difficulty with balance. Each 1-h session of the yoga program began with a warm-up (5–7 min) and ended with a relaxation period (10 min). To facilitate and guide home practice, participants were given an audio recording (CD) of the yoga instructions recorded by the yoga instructor. Group sessions were held twice per week, with 2-3 days between sessions. Participants were expected to practice yoga at home and record the number of minutes they engaged in yoga per day, but there was no certain amount or frequency required. The components of our intervention using Vinyasa style yoga are detailed in Table 1. Participants in the control group received general health-education materials mailed to their home every 2 weeks for 3 months, including content pertaining to risk factors associated with type 2 diabetes and the prevention of diabetes that included tips for healthy eating out, cardiovascular risk factors (hypertension and high cholesterol), consuming a balanced diet and engaging in physical activity. During the 3-month measurement, we discussed health-education content and responded to their questions and interpreted this to be a confirmation that they had reviewed the materials.

### 2.3. Measures

#### 2.3.1. Clinical Measures

BP, blood glucose, insulin, lipid levels [cholesterol; high-density lipoprotein (HDL); low-density lipoprotein (LDL); triglycerides] and body weight were obtained at baseline and at the end of 3 months. The blood test required a 12-h fast (no food or drink, except water and medications); *∼*20 ml of blood was obtained via a venipuncture in the arm with the individual in an upright position and after at least 5 min in a resting state. We measured weight (in kilograms) after an overnight fast with the participant dressed in light clothing and without shoes using the Tanita bioelectrical impedance scale (Tanita Corporation of America, Inc., Illinois, USA), and height (in meters) on a wall-mounted stadiometer. We calculated BMI using the equation of weight (kg)/height (m)^2^. All physical measurements were done at MUH-CTRC by their staff members who were blinded to group assignment. During the measurements, we were available to answer any questions and participants were asked not to discuss their program with MUH-CTRC staff. Participants in both groups were asked to describe their demographic and co-morbidity profile at baseline and complete questionnaires on exercise self-efficacy at baseline and at the end of 3 months. Those in the yoga group were asked to complete questionnaires on treatment expectation, program satisfaction and exercise log during or after the yoga program.

#### 2.3.2. Medical History and Sociodemographic Information

The Demographic Questionnaire and Brief Co-Morbidity Questionnaire, developed by the Center for Research in Chronic Disorders (CRCD) at the University of Pittsburgh School of Nursing, were used to collect data on age, marital status, race, educational level, employment status, income, living arrangements, religion, health insurance and co-morbidity at baseline.

Exercise self-efficacy [20, 23] was measured by the Exercise Self-Efficacy Scale at baseline and at the end of 3 months. Exercise self-efficacy is the degree of confidence one has in his or her ability to engage in exercise in the face of competing day-to-day conditions [20]. The Scale was developed by researchers at Stanford University to measure one's confidence in his or her ability to perform exercise routines regularly under various circumstances. It measures self-efficacy belief rather than outcome expectancy. The questionnaire consists of 18 items, each related to a different routine. The answers range from zero (“Cannot Do”) to 100 (“Certainly Can Do”) and are summed to yield a single total score. Higher scores on the scale indicate greater perceived self-efficacy to participate in physical activity. Cronbach's alpha for this scale was 0.95.

During the intervention (after Session 1, Session 8 and Session 16), treatment expectation was measured by the Treatment Credibility and Expectancy Questionnaire [24], which assesses perceived logic of and confidence in a treatment, willingness to recommend the treatment to a friend, and belief in the likelihood that the treatment will help their condition. Sum of the first three items (9-point Likert-type scale) gives the credibility score and the fourth item (10-point scale, 0–100%) provides the expectancy score. Higher scores indicate greater perceived treatment credibility and expectancy. The treatment credibility score had a high internal consistency coefficient (Cronbach's *α* = 0.86) in a study using cognitive-behavioral treatment for adults with panic disorder [25].

Acceptability of the yoga intervention was evaluated at the end of the yoga intervention by the Yoga Program Satisfaction Questionnaire. The questionnaire consists of five semi-structured questions related to participant satisfaction with the yoga program and feelings about the yoga program. In addition, each participant logged the average number of minutes he or she engaged in yoga practice per day in an Exercise Log. These self-reports provided a check on how much the participants were practicing yoga during the 3-month intervention period. Individuals were instructed to record daily to avoid recall bias.

### 2.4. Statistical Analysis

Data were analyzed using SPSS (version 15.0, SPSS, Inc., Chicago, IL, USA), with significance set at 0.05 for two-sided hypothesis testing. Demographic characteristics for each treatment group were described as means and standard deviations for continuous variables, and frequency counts and percentages for categorical variables. To examine the effect of yoga on the major outcome variables compared to the education group, the Mann-Whitney *U*-test was used to compare their mean differences between baseline and 3 months. Correlations (Spearman's rho) between attendance rate and outcomes were reported. Due to the small sample size in this pilot study, effect sizes (using Cohen's *d* comparing mean changes from 3 months to baseline between treatment groups) were also calculated to show magnitude and direction of the effect of the yoga intervention relative to the control condition for outcome variables. According to conventional definitions of effect size from a behavior science perspective [26], values of Cohen's *d* on the order of 0.20, 0.50, and 0.80 represent small, medium and large effects sizes, respectively. The sample size for this pilot study was determined considering the feasibility of recruiting and retaining study participants rather than to have sufficient statistical power to formally test hypotheses.

## 3. Results

All 23 participants finished the program and completed baseline and 3-month visits for the assessments and also completed the questionnaires.  Table 2 summarizes demographic characteristics of study participants. Ninety-one percent of the sample was female and 17.4% was minority. Their mean age was 51.7 years (SD = 4.9), and their mean years of completed education was 18.0 (SD = 4.1). The mean BMI was 29.79  ±  5.24 kg/m^2^ [28.2  ±  3.7 in the yoga group versus 31.5  ±  6.2 in the education group (*P* = .139)]. The average number of self-reported co-morbidities was 5.9 (SD = 3.6) with the most common co-morbidities reported being headache and depression. One-third of participants (*n* = 7, 30.4%) had pre-hypertension and the majority (*n* = 20, 87%) of them had abnormal cholesterol levels. All 12 participants completed the yoga program without complication; attendance ranged from 58.3 to 100% for the yoga sessions (mean  ±  SD 81.3  ±  14.9%). There was no significant relationship between attendance and amount of yoga practiced during the 3 months (mean  ±  SD 416.67  ±  492.52 min; range 0–1370 min).

Table 3 summarizes post-intervention changes in the major outcome variables. Compared to the education group, the yoga group revealed a pattern of improvements in weight (effect size *d* = −0.53), systolic BP (effect size *d* = −0.62), diastolic BP (*d* = −0.35), insulin (*d* = −0.34), total cholesterol (*d* = −0.22), triglycerides (*d* = −0.91) and exercise self-efficacy (*d* = 0.80). However, effect sizes for fasting glucose level (*d* = −0.20), LDL (*d* = −0.10) and HDL (*d* = 0.24) reflected small or no changes. Based on data from the Treatment Credibility and Expectancy Questionnaire, participants experienced increased confidence over time in recommending the yoga program to their friends at high risk for type 2 diabetes. There was a pattern for a positive relationship between attendance rate and perceived expectancy (Spearman's rho = 0.548, *P* = .065) for the yoga program, but no relationship was observed between attendance rate and perceived credibility (Spearman's rho = 0.206, *P* = .520). There were significant correlations between attendance rate and systolic BP (Spearman's rho = 0.651, *P* = .022). Participants in the yoga group indicated on the Yoga Program Satisfaction Questionnaire that they were highly satisfied with the program (mean  ±  SD 99.2  ±  2.9%; range 90–100%). They liked a slow and gentle yoga approach without peer pressure and reported that they gained strength, flexibility and balance through their yoga practice.

## 4. Discussion and Conclusion

This pilot study assessed the feasibility of implementing a yoga program among adults at high risk for type 2 diabetes. The preliminary study results indicate that this yoga program is feasible and acceptable to this population. Participants in the yoga group showed increased confidence over time in recommending the yoga program to their friends who are at high risk for type 2 diabetes and expressed high satisfaction with the program. Even though we did not use strategies to enhance adherence and retention for this pilot study, all participants completed the yoga program with an average attendance rate of 81.3%. Consistent with prior research [27, 28], individuals in the intervention group considered group interaction and support and the no-cost nature of our intervention to be worth the time commitment involved in attending the sessions.

As participants were asked not to change their exercise level by initiating any new form of exercise during this study, we limited our recommendations for physical activity to emphasizing the importance of being active in day-to-day life. Therefore, we do not have information on how yoga practice helps with adoption and maintenance of other physical activities. However, this pilot study helped us to understand the relationship between self-efficacy and practicing yoga. Individuals who practiced yoga showed a pattern of improvement in exercise self-efficacy, a strong and consistent predictor of adherence to multiple health-related behavior changes such as diet [29], physical activity [22] and smoking [30]. Therefore, we could hypothesize that if yoga could help enhance exercise self-efficacy, yoga practice may directly or indirectly change health-related behaviors such as adopting healthy exercise habits. Changed behaviors would influence physiological and psychological responses related to cardiometabolic risk factors and vice versa. Additional research would be necessary to determine how self-efficacy might be further enhanced with yoga practice as part of future development of interventions to promote exercise self-efficacy and to examine how yoga practice eventually leads to increased adoption of physical activity among adults at high risk for type 2 diabetes.

Compared with the education group, after the 3-month intervention, the yoga group experienced improvement in cardiometabolic risk factors. Small to large effect sizes were found in the between-group changes in cardiovascular risk factors such as weight, BP, insulin and triglycerides. Due to its small sample size, this pilot study did not duplicate the statistically significant results reported from previous studies [7–11]; however, this study will allow for the estimation of sample size in planning the next intervention study considering the targeted hypothesis testing of immediate and long-term treatment effects of yoga on cardiovascular risk factors. While the small sample limits the generalizability of our findings, the randomized, controlled trial design helps to better understand the benefits of the intervention program, because such a design permits allocation of participants that minimizes any bias from known and unknown determinants of outcome. Also, even though all physical measurements were done by MUH-CTRC staff members who were blinded to group assignment, we acknowledge a possibility of having exaggerated estimates of treatment effect due to participants' awareness of their control group assignment [31].

In summary, the results of this pilot study suggest that a yoga program could potentially be a risk reduction option for adults at high risk for type 2 diabetes. Anecdotal comments by study participants revealed that they perceived improvement in their strength, flexibility and balance after practicing yoga. We recommend that future studies involving yoga obtain objective measures of strength, flexibility and/or balance. Moreover, additional research with a larger sample and a longer follow-up for diabetes development is warranted to further evaluate the beneficial effects of yoga practice in this population.

## Funding

Central Research Development Funds (CRDF) of University of Pittsburgh and the Montefiore Clinical Translational Research Center (MUH-CTRC: NIH/NCRR/CTSA Grant UL1 RR024153).

## Figures and Tables

**Table 1:** Yoga sequencing.

Warm-up (5–7 min)	
Standing, seated or supine; includes all movements of the spine	
Sun-salutations (10–12 min)	
Full or modified, depending on the participant's needs	
Standing poses (12–15 min; poses are held for 3-4 breaths)	
Two-legged balances (warrior series, triangle series)	
One-legged balances	
Seated/kneeling poses and counter-poses (12–15 min; poses are held for 2-3 breaths)	
Spinal extension	
Hip-opener	
Spinal rotation; Spinal flexion	
Relaxation (10 min) using deep relaxation pose	

**Table 2:** Demographic information (*N* = 23).

	*n* (%)	Mean ± SD
Age (years)		51.7 ± 4.9
Gender		
Male	2 (8.7)	
Female	21 (91.3)	
Race		
White	19 (82.6)	
Black	2 (8.7)	
Hispanic	1 (4.3)	
Native American	1 (4.3)	
Level of education (years)		18.0 ± 4.1
Sedentary lifestyle	23 (100)	
Family history of diabetes	23 (100)	
Impaired fasting glucose	2 (8.7)	
Pre-hypertension	7 (30.4)	
Overweight/obese (BMI ≥30 kg/m^2^)	20 (87)	
Abnormal cholesterol levels	20 (87)	
Number of co-morbidities		5.9 ± 3.6

**Table 3:** Post-intervention changes in the major outcomes (*N* = 22).

									
	Yoga (*n* = 12)			Education (*n* = 10)			*z*	*P*-value	Effect size
	Baseline	3 months	Change	Baseline	3 months	Change			
	Mean (SD)	Mean (SD)		Mean (SD)	Mean (SD)				
Weight (Ib)	175.5 (30.0)	174.7 (29.8)	−0.79	188.4 (28.8)	189.4 (29.4)	1.02	−1.385	.166	−0.53
BP (mmHg)									
Systolic	119.3 (14.3)	114.1 (7.7)	−5.17	115.7 (15.9)	119.3 (13.9)	3.60	−1.519	.129	−0.62
Diastolic	68.7 (8.3)	69.3 (9.0)	0.58	68.9 (9.5)	72.6 (7.5)	3.70	−0.925	.355	−0.35
Fasting glucose level	90.1 (11.5)	92.8 (11.7)	2.75	91.2 (13.9)	95.8 (10.8)	4.60	−0.396	.692	−0.20
Insulin	10.5 (5.8)	8.1 (5.7)	−2.42	9.8 (5.3)	9.9 (7.4)	0.01	−0.799	.424	−0.34
Lipid panel									
Total cholesterol	209.8 (33.6)	193.3 (32.1)	−16.50	198.8 (46.5)	187.6 (34.1)	−11.20	−0.726	.468	−0.22
Triglycerides	120.5 (41.3)	109.0 (46.6)	−11.50	98.3 (48.1)	114.3 (47.9)	16.00	−1.914	.056	−0.91
LDL-C	130.3 (35.2)	117.8 (33.9)	−12.41	126.1 (46.8)	116.0 (32.2)	−10.10	−0.132	.895	−0.10
HDL-C	60.3 (10.1)	56.6 (12.8)	−3.75	57.0 (15.3)	51.4 (11.8)	−5.60	−0.298	.766	0.24
Exercise self-efficacy	43.9 (23.2)	57.9 (23.0)	13.97	52.5 (30.5)	49.2 (23.7)	−3.33	−1.583	.114	0.80

Change is from baseline to 3 months. One participant in the education group was excluded from data analysis because she was on special diet for weight loss.
